# Factors influencing dental care access in Jordanian adults

**DOI:** 10.1186/1472-6831-14-127

**Published:** 2014-10-17

**Authors:** Suhair Ref’at Obeidat, Amani Ghassan Alsa’di, Dafi Sultan Taani

**Affiliations:** Department of Applied Dental Sciences, Faculty of Applied Medical Sciences, Jordan University of Science and Technology, Irbid, Jordan; Department of Preventive Dentistry, Faculty of Dentistry, Jordan University of Science and Technology, Irbid, Jordan

**Keywords:** Dental care access, Regular dental attendance, Barriers, Adults, Survey, Jordan

## Abstract

**Background:**

The aims of this study are to assess the influences of demographic and personal factors on Jordanian adults’ abilities to use dental services and the barriers to regular attendance.

**Methods:**

A self-administered questionnaire was distributed to a sample of 650 Jordanian adults attending King Abdullah University Hospital (KAUH) (n = 200), Jordan University of Science and Technology–Dental Health Teaching Center (JUST–DHTC) (n = 150), Yarmouk University Health Clinics (YUHC) (n = 150), and United Nations Relief and Works Agency (UNRWA) clinics (n = 150). 614 questionnaires were completed. Statistical Package for Social Sciences (SPSS) was used to analyze data, generate descriptive statistics and perform multiple logistic regressions. The level of significance was set at α = 0.05.

**Results:**

Approximately 93% of participants were dental services users. 89% were irregular users, while only 11% were regular users. The main reasons given for not visiting dental offices regularly were lack of time (39.2%), cost of treatment (26.9%), treatment not needed (22.2%), and fear of dentists (15.1%). Only 6.7% of respondents stated that they had never visited a dentist, while more than half (56.1%) reported the lack of need for dental treatment as a major reason for not using dental services. Restorative therapy was the most frequently sought treatment (61.6%), while periodontal treatment was the least frequently sought (14.1%). Although respondents who were married and/or those having missing teeth were significantly more likely to use dental services, respondents who were single were more likely to be regular attendees.

**Conclusion:**

The overwhelming majority of adults were irregular attendees. Time and cost constraints, lack of need for treatment, and fear of dentists were reported as major barriers to regular attendance. The study findings call for planning of educational and promotional programs to increase Jordanians’ awareness of and regular use of available dental services to maintain health, which will be both socially and economically beneficial.

## Background

Access to high-quality dental care increases the quality and length of healthy life for everyone [1]. Access to oral healthcare is determined by the client’s ability to utilize and benefit from oral healthcare [2]. Access to dental care is important to enhance and maintain good oral health, because oral health is an integral component of general health. There is clear evidence that oral diseases, and particularly moderate and advanced periodontal diseases, can have a significant effect on systemic diseases and general health [3]. The goal should be to allow all people to receive the dental care they need regardless of their financial status, geographic location, or health status. This access is controlled by the degree of fit between the client and the health care system [4]. Access to health services is often measured not only by the supply of dental services [5], but also by the utilization of dental services [6]. Exploring these factors together produces more useful and integrated information on need, demand, and oral health outcomes.

In many developing countries, access to oral health services is very limited, while in developed countries and in some industrial countries, access to oral healthcare is much better [7]. In Australia, patterns of access to dental care are described using data collected in the 2002 National Dental Telephone Interview Survey (NDTIS 2002). This survey showed that 57.6% of the dentate adult population had made a dental visit in the last year. Older age groups, subjects with high income, and females were more likely to have visited a dentist in the last year. About 53% of the population reported that they visit a dentist for checkups rather than for dental troubles or dental pain. Younger age groups, females, and respondents with high income were more likely to visit for checkups [8]. Woolfolk et al. found that female gender, high socioeconomic status (SES), having dental insurance and middle age were potential factors of more frequent dental checkups [9]. Locker et al. also reported that those of low income level and those without dental insurance were more likely to report financial barriers to dental care [10].

In Jordan, few surveys have been conducted to assess dental access and attendance among Jordanians. The vast majority of Jordanian people visit the dentist only when they have a serious dental or oral health problem. “Treatment not necessary” and “cost” were found to be the common barriers to regular dental attendance. The most frequent treatments received by the respondents at their most recent visit were restorative therapy and tooth extraction [11–14].

As in most developing countries, Jordan has experienced a significant population increase. This change in demographics has strained dental services due to increased demand. Evidence from clinical practice and the few oral health surveys carried out in Jordan revealed that the prevalence of dental caries and periodontal diseases is high [12, 15, 16]. In Jordan, many people are at risk of oral diseases because of the poor use of dental services.

A PubMed literature search found few studies conducted to assess dental care access for Jordanians in general rather than a specific sub-topic, and none of them investigated this issue in detail. Therefore, the aims of the current study were to investigate the influence of demographic and personal factors among Jordanian adults on their ability to use dental services, and to explore the barriers to regular dental attendance. Survey findings will provide information related to citizens’ characteristics, behaviors, thoughts, and preferences associated with utilizing dental services. This information is essential to establish baseline data that may help in planning educational, promotional, and preventive programs to encourage the community to utilize dental services. By increasing dental visits, these programs may reduce or prevent pain, troubles, and consequences associated with oral, dental, and periodontal diseases, as well as aesthetic consequences that may negatively affect the quality of life of affected individuals. Furthermore, they may reduce the burden of costly oral, dental, and periodontal treatments.

## Methods

This cross-sectional study was conducted after obtaining approval from the Institutional Review Board (IRB) and Research Committees at Jordan University of Science and Technology (JUST). A self-administered questionnaire was distributed to a convenient sample of 650 Jordanian adults (18–65 years) attending KAUH (n = 200), JUST–DHTC (n = 150), YUHC (n = 150) and UNRWA Clinics (n = 150) after the investigators obtained their consent and explained the aims, importance, and method of the study. Upon their agreement, all participants signed the consent form that explains the study importance and procedure. The administrators of the sites where questionnaires were distributed approved arrangements for data collection. Only 614 questionnaires were completed and statistically analyzed. To protect the anonymity and confidentiality rights of the subjects and to increase the response rate, participant identification was not required.

The questionnaire, entitled “Access to dental care among Jordanian adults”, included two major parts. The first part contained questions related to participant demographics and personal data, including age, gender, marital status, residency, number of family members, educational level, occupation, monthly family income level, possession of dental insurance, type of dental insurance, number of missing teeth, general health status, and transportation difficulties. The second part included questions related to utilization of dental services, patterns of dental attendance among users, reasons of not visiting or irregularly visiting dentists, time elapsed since the most recent dental visit, treatment provided in that visit, and respondent preferences. A panel of faculty members from the faculty of Applied Medical Sciences, department of Applied Dental Sciences at JUST established the content validity of the self-administered questionnaire. A sample of 10 Jordanian adults were asked to complete the questionnaire on two separate occasions to establish test–retest instrument reliability.

### Data processing and statistical analysis

SPSS software version 11.0 (SPSS® Inc., Chicago, IL, USA) was used to generate descriptive statistics and analyze the data. Multiple logistic regression models were performed to detect statistically significant differences between the appropriate explanatory variables and dependent variables that represented utilization of dental services [users vs. non-users] and patterns of dental attendance (regular vs. irregular), using the backward stepwise Wald method (BSTEP). In this BSTEP, all appropriate variables were entered into the model. The independent variables specified in the variable list were then tested for possible removal from the model one by one at each step, based on the level of significance of the Wald statistic. The variable with the lowest p-value compared with PIN (0.05) was left in the model. If the significance level was greater than POUT (0.1) the variable was removed. The algorithm stopped when no more variables could be entered or removed. Odds ratios were generated and corresponding 95% confidence intervals (CI) calculated for all significant variables.

## Results

Prior to data collection, the questionnaire items were pre-tested by administering the questionnaire twice to the same 10 subjects within one week, demonstrating a high test-retest reliability of 92.5% agreement. Out of 650 questionnaires, 614 questionnaires were completed and returned by volunteers, giving a return rate of 94.5%. These participants ranged in age between 18 and 65 years. They were divided into three age categories (18–25, 26–45, and 46–65) for social and economic reasons. The majority (85.2%) were younger than 46 years. Slightly less than one half (45.9%) of the sample were males. About 40% of participants were single. Only 13.5% of study sample reported a family income above JOD 500 per month. About 57% of subjects had greater than secondary education. About 53% of participants were unemployed. 47.7% of study subjects reported that they did not have any missing teeth, and only 7.5% of participants reported that they had poor general health. Less than half (45.1%) of the sample reported that they faced transportation difficulties when visiting dentists (Table 1).

**Table 1:** **Sociodemographics and personal data of study sample** (**N** = **614**)

Variable	N (%)
**Age** **(Years)**	
18-25	246 (40.1)
26-45	277 (45.1)
46-65	91 (14.8)
**Gender**	
Male	282 (45.9)
Female	332 (54.1)
**Marital status**	
Single	246 (40.1)
Married	368 (59.9)
**Family income** **(JD/Month)**	
<250	309 (50.3)
250-500	222 (36.2)
>500	83 (13.5)
**Level of education**	
≤12 years	262 (42.7)
>12 years	352 (57.3)
**Occupation**	
Employee	289 (47.1)
Unemployed	325 (52.9)
**Reporting having missing teeth**	
No	293 (47.7)
Yes	321 (52.3)
**Reported general health status**	
Fair	568 (92.5)
Poor	46 (7.5)
**Transportation difficulties**	
No	337 (54.9)
Yes	277 (45.1)

Table 2 shows that greater than one half of participants had dental insurance. Less than one half (47.3%) had governmental insurance, and only 7.9% had private insurance. Table 3 shows that the majority (93.3%) of participants reported that they used dental services. 89.0% reported that they visited the dental office irregularly, only when they had dental trouble or when in pain. Few (11.0%) participants were regular dental attendees, visiting the dentist once every 6 to 12 months. The major reasons given by the subjects for not visiting dental offices regularly were lack of time (39.2%), cost of treatment (26.9%), treatment not needed (22.2%), and fear of dentists or dental treatment (15.1%). Only 6.7% of the study sample stated that they had never visited the dentist, while more than one half (56.1%) reported lack of need for dental treatment as a major reason for not using dental services.

**Table 2:** **Dental insurance data of study sample** (**N** = **614**)

Variable	N (%)
**Having dental insurance**	
No	261 (42.5)
Yes	353 (57.5)
**If yes,** **type of insurance**	
Governmental	167 (47.3)
Military	121 (34.3)
University	37 (10.5)
Private	28 (7.9)

**Table 3:** **Utilization of dental services and reasons for not attending dental office in the study sample** (**N** = **614**)

Variable	N (%)
**Utilization of dental services**	
Users	573 (93.3)
Non users	41 (6.7)
**Pattern of dental visits by users**	
Check up	63 (11.0)
On emergency/need	510 (89.0)
**Reasons for not attending the dental offices regularly**	
Cost (yes)	137 (26.9)
Time constrains (yes)	200 (39.2)
Fear from dentist or procedures (yes)	77 (15.1)
Treatment not needed (yes)	113 (22.2)
Difficulty to get appointment (yes)	36 (7.1)
Difficulty to reach clinic –distance (yes)	16 (3.1)
**Reasons for not using the dental services**	
Cost (yes)	7 (17.1)
Time constrains (yes)	4 (9.8)
Fear from dentists (yes)	1 (2.4)
Treatment not needed (yes)	23 (56.1)
Difficulty to get appointment (yes)	3 (7.3)
Difficulty to reach clinic –distance (yes)	1 (2.4)

Table 4 provides information related to the most recent dental visit. Less than one half (47.4%) of participants reported that they had visited the dentists during the last 12 months. Restorations (61.6%) were the most frequently sought dental treatments, followed by prostheses constructions (52.4%) and teeth extraction (34.6%). Periodontal treatment was the least (14.1%) required treatment. Table 5 shows subject preferences for dental clinic type. The majority (79.6%) of subjects prefer to visit private dental clinics for many reasons. The most frequently reported reasons were high treatment quality (66.1%), followed by lack of long wait times (46.2%) and the ability to complete the sought treatment (30.3%). The rest of the study sample reported that they preferred to visit public clinics for reasons related to cost of treatment and close proximity to the service location.

**Table 4:** **Last dental visit related data of study sample** (**N** = **614**)

Variable	N (%)
**Last dental visit**	
Less than one year	291 (47.4)
1–2 years	110 (17.9)
2–5 years	65 (10.6)
≥5 years	107 (17.4)
Never visited dentist	41 (6.7)
**Treatment provided in the last visit**	
Restorations (yes)	353 (61.6)
Prosthesis (yes)	300 (52.4)
Extraction (yes)	198 (34.6)
Periodontal treatment (yes)	81 (14.1)
Orthodontic treatment (yes)	94 (16.4)

**Table 5:** **Variables related to the preferred clinics by study sample** (**N** = **614**)

Variable	N (%)
**Preference of clinics**	
Public	125 (20.4)
Private	489 (79.6)
**Reasons for preferring public clinic**	
Low cost	79 (63.2)
Partially covered by insurance	49 (39.2)
Close location	22 (17.6)
**Reasons for preferring private clinic**	
Quality of treatment	323 (66.1)
No long waiting	226 (46.2)
Possibility to continue treatment	148 (30.3)
Availability of treatment type	69 (14.1)
Unavailability of dental insurance	78 (16.0)
Easiness to get close appointment	105 (21.5)

To determine the subjects’ characteristics regarding access to dental care, two main outcomes were investigated: utilization of dental services (users vs. non-users) and users’ pattern of dental attendance (regular vs. irregular). Multiple logistic regression analysis “main effect” models were calculated after entering the characteristics to be measured in the models. The independence of variables included in all regression models was tested and confirmed to ensure that the regression models could be appropriately interpreted.

The results of multiple logistic regression analysis are shown in Table 6. After entering all possible significant characteristics in the first logistic regression model, marital status and having missing teeth were identified as significant differences between dental service users and non-users. Participants were 6.6 times more likely to be dental service users if they had missing teeth. Married subjects were about three times more likely to be dental service users than singles. The odds of being a dental service user were 2.6 times higher among participants with fair general health compared with poor health, but this result was not significant. The second logistic regression model found that singles were about 82% more likely than married subjects to be regular as opposed to irregular attendees.

**Table 6:** **Multiple logistic regression models** – **utilization of dental services** (**R** ^**2**^ = **0.173**) **and users**’ **pattern of dental attendance** (**R** ^**2**^ = **0.05**) **for study sample**

Explanatory variables	Utilization of dental services (dependent)#	Users’ pattern of attendance (dependent)$
	User vs. not user (N = 614)	Regular vs. Irregular (N = 573)
	OR (95% CI)	OR (95% CI)
**Marital status**		
Married **vs.** Single	2.77** (1.3-5.8) 1.0	1.0 1.82* (1.07-3.1)
**Missing teeth**		
Yes **vs.** No	6.6*** (2.5-17.6) 1.0	Not significant variable
**Health status**		
Fair **vs.** Poor	2.6^NS^ (0.96-6.95)	Not significant variable

*Significant at 0.05 level. **significant at 0.005 level. ***significant at 0.001 level, *NS* = not significant.

The explanatory (independent) variables included in the model (Utilization of dental services)# were: Health status (Fair vs. Poor), Marital status (Married vs. Single), Missing teeth (Yes vs. No), Educational level (≤12 years vs. >12 years), Income level (<250 JD/Month vs. ≥250), Difficulties visiting (No vs. Yes), Having dental insurance (No vs. Yes), and Gender (Female vs. Male). Only Marital status and Missing teeth were significant factors influencing the utilization of dental services.

The explanatory (independent) variables included in the model (Users’ pattern of attendance)$ were: Health status (Fair vs. Poor), Marital status (Married vs. Single), Missing teeth (Yes vs. No). Marital status was the only significant factor influencing the users’ attendance pattern.

## Discussion

The overall finding of our survey was that 93.3% of respondents were dental services users. The majority visited dentists when they had trouble or pain, and only a few subjects were regular attendees. Our results are in agreement with the findings of other researchers [11–14, 17, 18] who found that the overwhelming majority of Jordanians, both adults and school children, visited dentists irregularly and for symptomatic reasons only, while only small numbers attended regularly for dental checkups or preventive reasons. The irregular utilization of dental services by Jordanians may be caused by their belief that dental conditions are not serious or life threatening, or may reflect their unawareness or lack of knowledge about the importance of visiting dentists for checkups and preventive reasons to maintain good oral health and to avoid oral and dental diseases.

In our study, the main reported reasons for not visiting the dentist regularly were “time constraints”, “cost”, “treatment not needed”, and “fear of dentists and dental procedures”. Among non-users, “treatment not needed” was the most frequent reason reported. These results are consistent with the findings of other researchers [11, 12, 17, 19]. A recent study found that “cost of services”, “fear of dentists”, “length of waiting lists”, and “availability of oral health care services” were the common factors for not using dental services among older adults in Australia [20]. The first two factors (cost and fear) were consistent with our findings, as were the results of a study reporting that the lack of perceived need for and awareness of the importance of regular dental attendance was the most frequent reason for dental avoidance among Europeans [21]. “Fear of the dentist” and “difficulty in obtaining a dental appointment” were the main reasons for irregular dental attendance reported by El-Qaderi and Taani [14]. Dental anxiety was also found to be an important factor for not seeking dental treatment by Crocombe et al. [22].

The most common treatments sought by our study subjects at their most recent dental visit were restorative therapy, followed by prosthesis and tooth extraction, while the least sought treatments related to periodontal health. Other researchers [12, 23] have also found that the most frequent treatment required by subjects at their most recent visit was restorative therapy.

This study illustrates that the majority of participants prefer visiting private clinics rather than public ones because of the high quality of dental treatment, short waiting times, and the possibility to continue treatment. Participants who preferred public clinics mentioned the low cost of treatment as the main reason for their preferences. This may indicate that the quality of treatment provided by public clinics is low due to the unavailability of dental equipment and materials and an imbalance in numbers between service users and service providers resulting from unplanned services allocations. Employing more dentists, dental hygienists, and dental assistants in the public sector may improve the quality of provided treatment.

None of the following demographic and personal characteristics were found to affect the use of dental services or the pattern of use: age, gender, family income, educational attainment, employment, reported general health, dental insurance, and transportation status. We found no statistical differences between age groups concerning the use of dental services or the regularity of their use. This is contrary to the findings of many conducted studies. Some researchers [23, 24] found that younger respondents were more likely to visit the dentist regularly for preventive reasons, while Woolfolk and his colleagues [9] found that middle aged participants had more dental checkups compared with the younger and older age groups. In our sample, both genders equally used dental services, and there was no difference between genders in the pattern of attendance. Al-shammari et al. [23] and Kakatkar et al. [24] found that females were less likely to visit dentist for preventive reasons due to dental fear, while Woolfolk et al. [9] found that females had more frequent dental checkups than males. Woolfolk et al. justify their finding by suggesting that females may utilize dental services more frequently, in spite of their fear, because they have a greater tendency to expect good outcome from dental attendance.

Our results did not reveal any variations in educational level between users and non-users of dental services. This means that highly-educated Jordanians are not aware of dental diseases and the importance of maintaining oral health. One possible explanation is that dental education is not incorporated in the country’s general education system, and people therefore do not have basic knowledge about oral health. Consequently, dental educational programs may be required to increase Jordanians’ knowledge about oral diseases and their prevention. This contradicts other studies [9, 23, 24] who found that educated respondents were more likely to visit a dentist regularly for checkups or prevention, and who emphasized that an increase in educational attainment will decrease the barrier to regular dental visits.

The current study found that income level was not correlated with individual use or pattern of use of dental services. This finding corresponded with Al-shammari et al. [23], who stated that finances were not considered by respondents to be a barrier to using dental services, because dental care is provided free of charge for Kuwaitis, which is not the case in most countries. However, many researchers [10, 25–28] found that low income level respondents were less likely to use dental services and were often less satisfied with treatment they received. Woolfolk et al. [9] also found that income levels affected access to dental care, and that people with an income of $70,000 or more had more dental checkups than people at a lower income level. Kakatkar et al. [24] found that while the higher income group in their sample had better access to dental procedures than the lower income group, income was inversely correlated with dental visit frequency. They proposed that this is caused by social and cultural beliefs.

Although our study confirmed that respondents who were married were more likely to be dental service users than with respondents who were single, respondents who were single were more regular users. This may partially be because the unmarried respondents can use dental services for checkups and prevention more frequently, because they have more available time or fewer responsibilities. Dental insurance was not found to be a motivating factor for utilizing dental services in our sample, which is consistent with the finding of Halasa and Nandakumar [29] and inconsistent with the results of other researchers [9, 10, 30, 31] who found that people with dental insurance were more likely to have dental checkups and dental visits than those who were uninsured.

As expected, our study found that people with missing teeth were more likely to use dental services than those without missing teeth. This may be because the majority of Jordanians use dental services when they have trouble or pain rather than for checkups or prevention. Furthermore, respondents with missing teeth are less concerned with maintaining their teeth, so they visit the dentist for restorative, constructive, and surgical reasons. This finding contradicts the results of a survey conducted in an Australian population [32].

Finally, respondents who reported fair general health were not significantly more likely to be dental service users than those with poor health. This may signify that having disabilities, general health problems, or diseases may affect access to dental care.

The comparability of our results with those of other studies may be limited by differences in cultural and economic factors, subjectivity in answering questions, social variations among studied populations, and also differences in objectives, criteria, methodologies, and sampling techniques and sizes. Increased funding for research would facilitate increasing the number of data collectors and expanding the coverage of such research to cover the whole country. Consequently, the sample size, collection method, and localization of the study to a particular governorate were considered the main limitations of this study, meaning that the findings could not be generalized for all Jordanian adults.

## Conclusions

Use of dental services and pattern of use can serve as indicators of oral health related behaviors and beliefs. The overwhelming majority of Jordanians were irregular attendees and used dental services when they had dental trouble or pain, rather than for prevention and checkups. “Time constraints”, “cost”, “treatment not needed”, and “fear of dentists and dental procedures” were reported as major barriers causing irregular attendance, and they indicated Jordanian adults’ low perceived need to use dental services on a regular basis for checkups and prevention.

### Clinical significance

Our study findings call for planning of educational and promotional programs to increase Jordanians’ awareness of the need to use available dental services regularly to maintain socially and economically productive life and to reduce the burden of costly dental treatments. To increase regular dental attendance, barriers must be controlled through appropriate education and intervention. To motivate people successfully, it is necessary not only to provide information but also to pay attention to the individual factors that restrict their behaviors. Nationwide studies should be conducted to generalize these findings to the whole country and to study further potential factors that may affect access to dental services, including psychological factors, general health status, emotional factors, social factors, and also assessment of provided dental services and assessment of dental team professionals.
